# Eradication of CML stem cells

**DOI:** 10.18632/oncoscience.327

**Published:** 2016-11-23

**Authors:** Bing Z. Carter, Michael Andreeff

**Affiliations:** Section of Molecular Hematology and Therapy, Department of Leukemia, The University of Texas MD Anderson Cancer Center, Houston, TX 77030, USA

**Keywords:** CML, stem/progenitor cells, TKI, Bcl-2, blast crisis

Bcr-Abl tyrosine kinase inhibitors (TKIs) have become the standard of care for patients with chronic myeloid leukemia (CML). Indeed, patients experience high response rates and long-term survival with continuous TKI treatment. However, TKIs rarely cure CML due to their inability to target CML stem cells. Consequently, CML will soon become the most prevalent leukemia with 100,000 patients in the U.S. alone. Long-term treatment with TKIs is extremely expensive, associated with side effects, and development of resistance in some patients. Resistance can fuel the progression to blast crisis (BC), which is associated with almost complete chemo-resistance and extremely poor treatment outcome.

During the last decade, significant insights into CML stem cell biology and mechanisms of TKI resistance were gained leading to the development of combinatorial strategies to target CML stem/progenitor cells and to overcome TKI resistance [1,2]. We and others have established Bcl-2 family proteins as key apoptosis regulators and specifically anti-apoptotic Bcl-2 proteins as crucial survival factors for myeloid leukemia cells and stem/progenitor cells. Inhibition of anti-apoptotic Bcl-2 proteins with dual Bcl-2/Bcl-xL or pan-Bcl-2 inhibitors was shown to target CML stem/progenitor cells and enhance the therapeutic efficacy of TKIs [3,4].

The tumor suppressor p53 regulates apoptosis primarily by transcriptional activation of pro-apoptotic Bcl-2 family proteins. Although frequently mutated in solid tumors, p53 mutations are rare in CML. We demonstrated that the activation of p53 via inhibition of its negative regulator, MDM2, in combination with TKIs synergistically targeted quiescent CD34+ BC CML cells [5], and Holyoake recently reported that dual targeting of p53 and c-MYC selectively eliminated CML stem cells [6].

To improve specificity and efficacy, and minimize toxicity, it is important to recognize which Bcl-2 proteins are indispensable for CML stem cell survival. Until recently, most Bcl-2 inhibitors were relatively non-specific and targeted several Bcl-2 proteins. Furthermore, our knowledge of the expression of Bcl-2 family members in hematopoietic and CML stem/progenitor cells is essentially limited to RNA, not protein levels, primarily because stem/progenitor cells account for only a very small portion of total bone marrow (BM) cells.

CyTOF (“cytometry by time-of-flight”) combines mass spectrometry and flow cytometry and constitutes a novel single cell proteomics system that can determine the expression of currently over 40 (potentially 120) cell surface and intracellular proteins simultaneously without the spectral overlap, and therefore able to determine the expression of multiple proteins/phosphoproteins in a phenotypically well-defined cell population. Using CyTOF, and an inducible transgenic chronic phase CML mouse model (Scl-tTa-BCR-ABL), we determined the protein levels of Bcl-2 family members in total BM and the very rare primitive BM LSK (Lin-cKit+Sca-1+) cells of CML (Tet-off) and control (Tet-on) mice. We found an overall increase of Bcl-2 and Mcl-1 levels in BM CD45+ cells, and of Bcl-2, Mcl-1, and Bcl-xL in LSK cells from CML mice compared to normal control mice. Notably, we found that only the Bcl-2 protein levels were higher in the LSK compared to total CD45+ cells in CML mice, but not in control mice [7]. We then treated CML mice with the specific Bcl-2 inhibitor ABT-199 (venetoclax) or with ABT-199 plus the Bcr-Abl TKI nilotinib and observed that selective inhibition of Bcl-2 greatly prolonged survival of CML mice and reduced leukemic LSK cells, which was significantly enhanced when combined with nilotinib. Importantly, the combination was highly effective in preventing secondary engraftment and reducing leukemia stem cell frequency (>20 fold compared to controls) [7], suggesting eradication of leukemia stem cells. Furthermore, and relevant to human disease, ABT-199 synergized with TKIs in the induction of apoptosis in CD34+CD38-, CD34+CD38+, and quiescent CD34+ stem/progenitor cells from BC CML patient samples [7]. This work is highly significant because it illuminates Bcl-2 as a key survival factor for CML stem cells and the potential of combinations of a Bcl-2 inhibitor and TKIs to eradicate CML stem cells and cure the disease. Importantly, selective inhibition of Bcl-2 avoids the massive thrombocytopenia, which is a serious side effect due to the inhibition of Bcl-xL by dual/pan Bcl-2 inhibitors in megakaryocytes and platelets. Furthermore, ABT-199 is FDA approved for a subset of chronic lymphocytic leukemia patients, and is under clinical evaluation for other hematological malignancies. Its major side effect in clinical trials is granulocytopenia, which parallels the activity against chronic myeloid (=granulocytic) leukemia.

In addition, β-catenin cross-talks with Bcr-Abl signaling and plays critical roles in leukemogenesis and stem cell function. Specifically, activation of β-catenin signaling promotes BC transformation and confers stemness to BC CML GMP progenitors [8]. We demonstrated that β-catenin and Bcr-Abl signaling proteins are highly expressed in BC CML progenitors, and that the combined inhibition of these proteins selectively targeted β-catenin-overexpressing BC CML progenitors *in vitro* and *in vivo* and prolonged survival of TKI-resistant human BC CML xenografts (manuscript in preparation).

**Figure 1:**
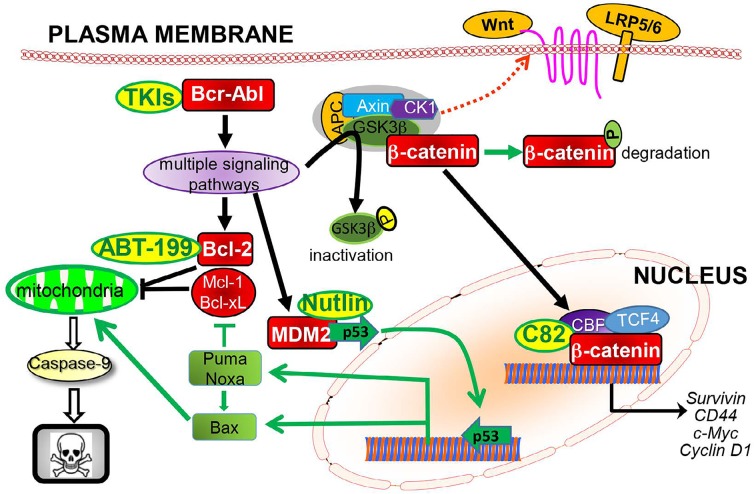
Bcr-Abl activates multiple survival signaling pathways that regulate Bcl-2, β-catenin, and MDM2, and combinations of TKIs with agents selectively targeting Bcl-2 (ABT-199), β-catenin (C82), or MDM2 (nutlin) synergistically induce apoptosis in CML cells and CML stem/progenitor cells.

Clearly, potentially curative therapies for CML require combinations of TKIs with other targeted agents, including those inhibiting Bcl-2 and β-catenin and activating pro-apoptotic TP53 signaling, all of which are important for stem cell function and are intertwined with Bcr-Abl signaling in CML cells as illustrated in Figure 1. Considering the heterogeneity of leukemia cells and stem cells observed within and between individual patients, particularly in those with disease progression, we envision that a deeper understanding of CML stem cell biology, coupled with the discovery of additional regulators of CML stem cell survival and self-renewal, will yield therapeutic options of combining multiple targeted therapies to achieve more effective leukemia stem cell eradication, prevent/overcome drug resistance, alleviate toxicity, and ultimately cure patients with CML.
